# The Efficacy of School-Based Interventions in Preventing Adolescent Obesity in Australia

**DOI:** 10.3390/healthcare8040514

**Published:** 2020-11-25

**Authors:** Kakale Buru, Theophilus I. Emeto, Aduli E. O. Malau-Aduli, Bunmi S. Malau-Aduli

**Affiliations:** 1 College of Medicine and Dentistry, James Cook University, Townsville, QLD 4811, Australia; kay.buru@my.jcu.edu.au; 2 College of Public Health, Medical and Veterinary Sciences, James Cook University, Townsville, QLD 4811, Australia; theophilus.emeto@jcu.edu.au (T.I.E.); aduli.malauaduli@jcu.edu.au (A.E.O.M.-A.)

**Keywords:** adolescent obesity, Australia, school-based interventions

## Abstract

Current trends suggest that adolescent obesity is an on-going and recurrent decimal that is still on the rise in Australia and the social burden associated with it can significantly cause low self-esteem and lack of confidence in personal body image in adulthood. Nonetheless, evidence-based prevention programs are not widely implemented in schools, even though they are commonplace for easy access to adolescents. The primary objective of this systematic review was to assess the scope and efficacy of adolescent obesity intervention strategies in Australian schools, to guide future research. Seven electronic databases were searched for peer-reviewed school-based intervention articles written in the English language and targeting 12–18-year-old adolescents. Intervention characteristics were extracted, and quality, efficacy and outcome measures were assessed utilizing thirteen studies that met the inclusion criteria for this review. Most of the Australian adolescent obesity research emanated from the State of New South Wales and none were nationwide. Five studies successfully met all the requirements in all measured outcomes, four met at least one measured outcome and the remaining four were unsuccessful. Despite the weak evidence of intervention efficacy for most of the reviewed studies, school-based interventions with multi-component combinations of physical activity, nutrition and alignment to a theory yielded promising results. Our findings point to the need for future research to assess the perceptions of school stakeholders in relation to the barriers and enablers to establishing school-based prevention and intervention programs for adolescents.

## 1. Introduction

Obesity (the abnormal or excessive accumulation of fat in the body) in adolescents continues to be a subject of increasing global public health importance, highlighting the need for evidence-based public health action towards its prevention and control [1,2]. Adolescence represents a period marking the transition from childhood to adulthood and classically covers the ages ranging from 12 to 18 years [3]. Obesity increases the health risks of individuals and is a major contributor to chronic disorders such as diabetes, cancer and cardiovascular diseases [4,5,6,7,8,9]. While excessive food intake and sedentary lifestyles are the main causes of obesity [10], other factors such as medical illness, use of certain medications, consumption of energy-dense foods or beverages and eating disorders, especially, binge eating, have also been associated with the risk of the disorder [10,11]. Furthermore, the social burden associated with obesity in adolescence can significantly cause low self-esteem and lack of confidence in personal body image in adulthood [12].

Over the past three decades, the prevalence of overweight and obesity in adolescents has continued to increase in several regions and countries around the world [12]. According to the World Health Organization [13], 340 million children and adolescents [12] were overweight or obese in 2016 [13]. This problem is of particular importance in Australia because even though the prevalence of childhood obesity has plummeted in the last two decades [14], this is not the case among adolescents [15]. Australia currently ranks as one of the top ten nations with the highest proportion of overweight or obese adolescents [16]. For example, Abaca-Gómez and colleagues [12] reported that in Australia, between 2010 and 2015, obesity in adolescent boys increased from 5.1% to 5.7% while in girls it increased from 4.2% to 5.4%. The authors suggested that abdominal obesity, which is a marker for cardiometabolic risks significantly increased from 7.2% to 15.8% and 3.6% to 8% in high school adolescent boys and girls, respectively, during the same period.

In the absence of effective preventive interventions, the prevalence of obesity in adolescents is projected to increase exponentially to 91 million by the year 2025 [17]. In 2016, the Australian Bureau of Statistics (ABS) reported that over a third of adolescents aged between 12 and 17 were overweight and obese [18]. This premise underscores the importance of a prompt public health response to the growing global burden of obesity among adolescents. Available evidence indicates that the prevailing Covid-19 pandemic exacerbates some of the main causes of the obesity such as physical inactivity and increased screen time [19]. The pandemic is also causing rapid weight gain in most regions of the world; this relationship between Covid-19 and obesity has been recently named “Covibesity” [20]. For instance, in a recent China-based study, physical activity time was estimated to have decreased from 540 min per week before the Covid-19 pandemic to 105 min per week during the pandemic—a reduction of over 430 min of physical activity time per week [19]. The study further revealed that the prevalence of physical inactivity has risen from 21.3% to 65%, while screen time has increased to 30 h per week. Similar findings were reported in a recent survey conducted in 298 schools (making up to 1.6 million adolescents aged 11 to 17 years) in 146 countries worldwide [1]. This evidence further highlights the need for urgent actions against the obesity epidemic.

In Australia, guidelines aimed at improving the health and wellbeing of children and adolescents through regular participation in physical activities and dietary recommendations (vegetables, fruits, etc.) are in place [21]. However, a substantial proportion of adolescents in the country often do not adhere to these guidelines [6,22]. For example, the 2018 report card for “The Active Healthy Kids Australia” (AHKA) indicated that Australian adolescents fell below the set targets for active transport, sedentary behavior and physical activity [22]. Guthold, Stevens, Riley and Bull [1] also indicated that Australia had the highest prevalence of physical inactivity among adolescents—precisely 89%—followed by New Zealand in the category of high-income Western Countries. Given the fact that regular physical activity and the practice of healthy diets are critical to shedding excess weight or maintaining a healthy weight, with a range of other health benefits [23], it is important to investigate better means of addressing the obesity epidemic among Australian adolescents.

Given that the majority of adolescents are in high school [24], schools represent one of the most ideal environments for implementing dietary and physical activity intervention programs aimed at addressing the obesity epidemic in the country [25]. Clarke and colleagues [24] posit that schools have the capacity to reach the majority of young people frequently and for longer periods. In addition, many school stakeholders are known to have been directly involved in implementing programs aimed at preventing obesity either as part of their duty or through the curriculum [24]. Stakeholders such as teachers of Home Economics, Science, Health and Physical Education, Dance and pastoral care advisors, health personnel and senior management have formal and informal access to many young people within the school environment, hence they can facilitate discussions on health topics such as body image, nutrition and weight control [26]. Nonetheless, evidence-based prevention programs are not widely implemented in Australian schools, even though they are commonplace for easy access to adolescents [22]. The prevalence of adolescent obesity in Australia warrants a review of the efficacy of the intervention programs to identify what the challenges are.

Consequently, the objectives of this systematic review were to (1) identify the scope of Australian school-based intervention strategies aimed at preventing adolescent obesity and (2) assess the efficacy of the interventions. The findings will be fundamental for the provision of a roadmap to necessary adjustments and developments to be effected in alignment with programs and interventions that have demonstrated significant results in reducing the prevalence of obesity in the past, an area where more evidence is needed. Furthermore, the first objective of the review will allow for scoping and synthesis of existing research evidence on school-based interventions, help identify gaps in the literature and give directions for future research.

## 2. Materials and Methods

This systematic review was conducted and reported in accordance with the Preferred Reporting Items for Systematic Reviews and Meta-Analyses (PRISMA) Statement [27].

### 2.1. Inclusion and Exclusion Criteria

In this review, the criteria for selection were: (1) peer-reviewed quantitative studies conducted in Australia from 2009 and current, (2) studies targeting adolescents (12 to 18 years old) attending an Australian high school or college, (3) studies where interventions were implemented in a school setting, (4) studies published in the English language and (5) community interventions for adolescents implemented by school stakeholders in a school setting.

The criteria for exclusion were (1) studies targeting children below the age of 12 and young/mature adults above the age of 18, (2) intervention studies targeting adolescents but were implemented out of a school setting and (3) studies not published in the English language.

### 2.2. Search Strategy

For this review, peer-reviewed articles published in the last 10 years in English and indexed in the following databases were searched: Eric, PubMed, InFormit, Scopus, CINAHL, Med-OVID and Psych Info. Google Scholar and included studies were also screened and hand searched for relevant additional inclusions.

### 2.3. Search Terms

The following search terms were used during electronic database searches: Obesity OR obes* OR overweight OR chubby OR plump OR plump OR plus size OR body fat OR excess adiposity OR body mass index, AND School personnel OR high school OR school role OR school organization OR school schedule OR school involvement OR school health services OR school policy OR school effectiveness OR high school, AND Program OR intervention OR initiative OR prevent OR solution OR scheme OR strateg* OR project OR program development, OR strategic planning OR early intervention OR program evaluation OR intervention OR program effectiveness OR program success OR program results, AND High school students OR student OR youth OR adolescent* or teenage*.

### 2.4. Data Extraction

Two authors (K.B. and B.S.M.-A.) identified and independently screened the titles and abstracts of the retrieved articles. The articles which did not meet the inclusion criteria were removed. Full-text articles categorized as potentially eligible for inclusion were subsequently screened by both authors in a consensus meeting and disagreements were resolved in real time until a consensus was reached. Study specific information from the included studies was extracted and these included: the publication year, study design, study location and duration, study participants, type of intervention and reported outcomes. The other two authors (T.I.E. and A.E.O.M.-A.) validated the data.

### 2.5. Risk of Bias Assessment

The Quality Assessment Tool for Studies with Diverse Designs (QATSDD), a 16-item tool which allows for comparison of studies with different research method designs, was used to assess the quality of included studies [28]. The tool was modified to exclude 2 items: “Fit between research question and format and content of data collection method (Qualitative only)” and “Assessment of reliability of analytical process (Qualitative only)” as they were not applicable to this study. The criteria included were: (1) theoretical framework; (2) aims/objectives; (3) description of research setting; (4) sample size; (5) representative sample of target group; (6) procedure for data collection; (7) rationale for choice of data collection tool(s); (8) detailed recruitment data; (9) fit between research question and method of data collection (quantitative only); (10) fit between research question and method of analysis; (11) good justification for analytical method selected; (12) strengths and limitations; (13) evidence of user involvement in design and (14) statistical assessment of reliability and validity of measurement tools. A scale of 0–3 (not at all = 0, very slightly = 1, moderately = 2 and complete = 3) was used to assess the quality of each criterion item of assessment. All scores for each criterion were added together and converted into percentages for easier interpretation. Scores less than 50% were classified as low quality and scores of 50%–80% and over 80% were classified as medium and high quality, respectively.

### 2.6. Data Synthesis

#### Study Selection

**Figure 1:**
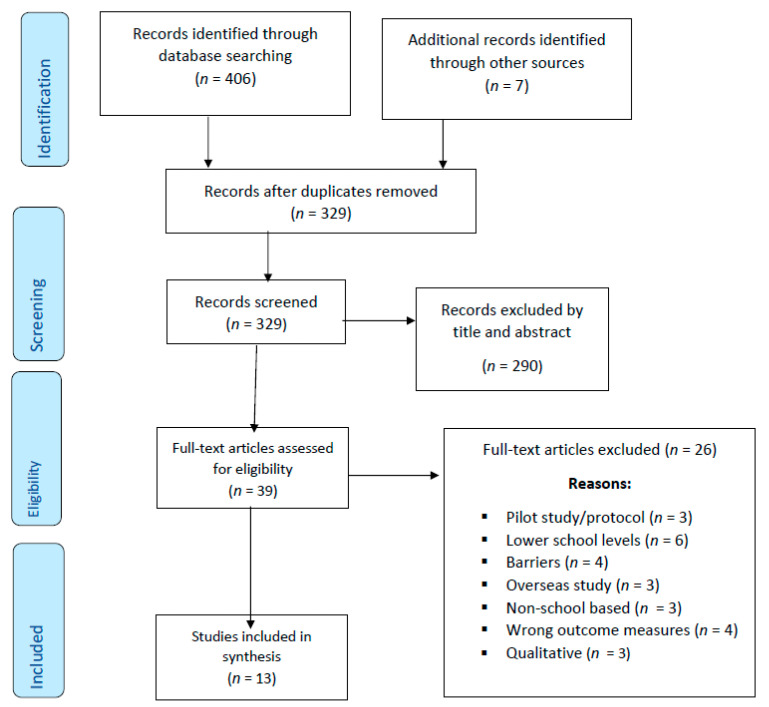
Preferred Reporting Items for Systematic Reviews and Meta-Analyses (PRISMA) Flow Chart.

An initial search identified 413 papers, including seven hand searched ones. After removing duplicates, screening titles and abstracts, 39 papers remained for full-text review with thirteen included in the systematic review. The PRISMA flow chart for the review is shown in Figure 1.

## 3. Results

### 3.1. Characteristics of Included Studies

Table 1 displays the characteristics of the included studies. The studies were conducted in three Australian states and the capital territory: New South Wales (NSW) had nine studies (*n* = 9), Victoria [18] had two studies (*n* = 2), Queensland (QLD) had one study (*n* = 1) and one study was conducted in the Australian Capital Territory (ACT). Of the 13 included studies, ten were randomized controlled trials, while three were quasi-experimental longitudinal studies. The sample sizes ranged from thirty-three (*n* = 33) to two-thousand and fifty-four (*n* = 2054). The number of participating high schools per study ranged from one (*n* = 1) to fourteen (*n* = 14). Eight studies (*n* = 8) had only boys as participants, one study (*n* = 1) had only girls as participants and the remaining five (*n* = 5) studies had both boys and girls as participants. The age range of the participants was 12–17 years. The studies with the longest duration lasted for three years while the shortest duration was approximately four months.

### 3.2. What Types of Interventions Were Identified?

Table 2 shows the interventions reported in the included studies. There were three intervention types identified: physical activity, sedentary behavior and nutritional behavior. Of these three types of interventions, physical activity was used in 11 of the 13 reviewed studies, followed by sedentary behavior, which was used in nine studies. Six studies combined all the three types of interventions [29,30,31,32,33,34]. None of the studies utilized nutritional behavior only. All reviewed studies were Randomised Controlled Trials (RCTs) and quasi-experimental research designs.

### 3.3. Health Outcomes Reported

The reviewed articles reported on adiposity, physical activity, behavioral and nutrition outcomes, as shown in Table 2 above. Adiposity- and weight-related outcomes were waist circumference, weight, lean tissue mass and percent body fat. Physical activity included organized sport, standing instead of sitting, jumps and active transport. Behavioral outcomes included screen time while nutrition outcomes included fresh fruit and vegetable intake and sugar-sweetened beverage intake. Twelve studies [30,31,32,33,34,35,36,37,38,39,40,42] had BMI outcomes while five studies had a BMIz-score outcome. Six studies [31,32,33,37,39,40] measured multiple outcomes across adiposity, physical activity, sedentary behavior and nutrition outcomes.

### 3.4. Intervention Efficacy on Measured Outcomes

#### 3.4.1. Changes in BMI and BMIz

Overall, out of the 12 studies [30,31,32,33,34,35,36,37,38,39,40,42] that reported on BMI, only four studies recorded a significant reduction in BMI in the intervention group in comparison to the control [31,32,34,36], whereas eight studies [30,33,35,37,38,39,40,42] recorded no significant changes in BMI between the intervention and the comparison groups at baseline, during the intervention and the end of the intervention. Of the five studies that reported on BMIz, three studies [31,33,36] recorded significant reductions in BMIz in favor of the intervention group.

#### 3.4.2. Changes in Other Adiposity and Weight-Related Outcomes

Of the nine studies [29,30,32,33,34,38,39,40,42] which measured the effect of the intervention on body fat, three studies [30,34,41] reported a significant reduction in body fat for the intervention group in comparison to the control group where no significant changes were reported, while five studies [32,33,38,39,40] reported no significant differences in body fat for both the intervention and control groups. Four studies measured waist circumference [31,37,39,40], but reported no statistically significant differences between the intervention and the control groups.

Participants’ body weight was measured in five studies [31,32,33,36,42]. One study [33] reported a significant reduction in body weight for the intervention group as compared to the control group. A moderate reduction in body weight was recorded in two studies [32,36] both at baseline and at the end of the intervention in favor of the intervention group, while the remaining two studies [31,42] reported no significant reduction in body weight. Only one study [41] measured lean tissue and participants in the intervention group indicated a significant gain in lean tissue compared to the control group. Only one study [35] measured and recorded a significantly higher energy expenditure among participants in the intervention group compared to the control group.

#### 3.4.3. Changes in Physical Activity

A total of nine studies [30,31,32,33,34,37,39,41,42] reported on active transport and physical activity-related outcomes. Two studies [33,34] recorded a significant increase in active transport in the intervention groups in comparison to the control groups where no changes were seen. One study recorded an increase in physical activity during recess only but no impact on active transport for both the intervention and control groups [32], whereas in another study [39] only 50% of the participants attended physical activity sessions during recess. One study [34] reported significant changes in multiple physical activity-related psychological outcomes, such as a significant increase in physical self-worth, perceived physical condition, resistance training self-efficacy and physical activity behavioral strategies, in favor of the intervention group compared to the control group. The remaining five studies [30,31,37,39,42] did not record any significant increase in physical activity for both intervention and control groups. However, one study [42] reported that though there was no comparative effect between the intervention and the control groups, boys performed better than girls within the intervention group in moderate-to-vigorous physical activity at 12-month follow-up and the intervention group spent significantly more time in vigorous activities each day.

#### 3.4.4. Changes in Sedentary Behaviors—Screen Time

Screen time was commonly measured by six studies [30,31,33,34,37,40] but yielded diverse results. Three studies [31,37,40] reported significant reductions in screen time and compliance with daily recommended restrictions for the intervention group whereas screen time increased for the control group. On the contrary, two studies [32,33] reported a significant drop in screen time in favor of the control group, while screen time increased significantly for the intervention group, exceeding the daily recommendation of not more than two hours. The last study [30] recorded no changes in screen time for both the intervention and control groups.

#### 3.4.5. Changes in Nutritional Behaviors

Six studies [31,32,33,37,39,40] reported on nutritional behaviors, particularly sugar-sweetened beverages, fresh fruit and vegetables, whereas two studies [31,40] reported a significant reduction in the intake of sugar-sweetened beverages, fresh fruit and vegetables for the intervention group while there was no change with the control group. In one study [32], even though there were no significant changes in the school tuck-shop menu and role modeling from teachers, the intervention group showed a significant increase in the awareness of healthy eating habits compared to the control group where there were no changes observed. None of the studies reported a significant increase in the intake of fresh fruit and vegetables for any of the groups. Two studies [33,39] reported no significant changes for the intake of sugar-sweetened beverages and fresh vegetables for both intervention and control groups. One study [37] reported no significant changes in the intake of sugar-sweetened beverages for both groups.

### 3.5. Theoretical Model

Social cognitive theory was predominantly used as a guide in developing intervention components; 9 out of the 13 reviewed studies [30,31,32,34,36,37,39,40,41] utilized this theory. A social ecological model was used in two studies [40,41], one study [36] used a combination of three models; self-determination theory, socio-ecological theory and health promoting schools framework. Four studies [36,37,40,42] combined more than one theoretical model. One study [33] used a community-based capacity building approach. Three out of the 13 selected studies [35,38,42] did not indicate any link to a guiding framework or theory.

### 3.6. Quality Appraisal

The QATSDD assessment shown in Table 3 indicated that the scores ranged from 55% to 83%. Eleven studies [29,31,32,33,34,35,37,38,39,41,42] were of a medium quality. Two studies [30,36] were of a high quality. One study [38] had the lowest percentage (55%) within the medium quality-rated studies because it lacked a theoretical framework, did not provide a rationale for choice of data collection and had a poor description of strengths and limitations of the study. Given that all reviewed studies were RCTs and quasi-experimental research designs, they were judged to be appropriate because they provide a high level of reliable evidence.

## 4. Discussion

In this review, we identified 13 school-based intervention studies which were conducted in Australian high schools. By focusing only on studies investigating school-based interventions and excluding those that combined school-based interventions with home- and community-based interventions, we are able to ascertain that the efficacy of interventions were as a result of school-based interventions [43]. The intervention components included physical activity, dietary and sedentary behaviors such as reducing screen time. The interventions from the included studies varied in their duration, the ages of adolescent participants, sample size and the main outcome variables; hence, we were unable to conduct a meta-analysis. Evidently, NSW and Victoria have been found to be more proactive in participating in school-based interventions in this review. Future research studies should consider investigating reasons for minimal to non-participation in other states in school-based intervention toward adolescent obesity.

Overall, of the 13 reviewed studies, five studies [31,32,33,34,36] demonstrated successful implementation of the intervention across all measured outcomes, four studies [30,35,40] reported significant results on at least one of the measured outcomes while the remaining four studies [37,38,39,41] had no significant results across all measured outcomes. The studies that reported successful outcomes combined physical activity and nutrition behaviors and used one or more theoretical frameworks. The findings from this review corroborate findings from previous systematic reviews that analysed the efficacy of obesity interventions [34,44]. For instance, Chen and Wilkosz [44] reported that all the successful interventions in their review incorporated both dietary and physical activity strategies as components of the intervention and had alignment with a theory. Another noteworthy finding is that one of the successful studies [34] also incorporated physical activity-related psychological outcomes. The findings suggest that incorporating physical activity, dietary components and psychological incomes in future interventions could possibly reduce adolescent obesity [44,45]. The success of these interventions could potentially be because schools were given financial support to develop an enabling environment for the promotion of good nutrition and physical activities [32]. Participation in the interventions was also made free of charge for the participating schools and students [31,32]. Success rates for these interventions can be attributable to initiatives such as the recognition of participants through certificate awards [31,34], new fitness equipment purchased for the schools, removal of vending machines and school food and water policy introduction [33]. These findings highlight the importance of a multi-pronged approach that involves opportunities for students and a supportive ecosystem in combating adolescent obesity problems. All studies underscore the need to identify barriers to the efficacy of school-based obesity prevention interventions and develop targeted strategies to achieve a more generalizable outcome.

The poor outcomes from the studies [37,38,39,42] that did not yield any noteworthy outcomes have been linked to intervention activities being offered during lunch breaks when there are other competing interests for students. One study indicated that only half of the students attended these sessions [39]. The duration of the interventions may also affect their outcome. For example, two studies [39] indicated that interventions only lasted for 6 months while in the other two [37,42], it lasted for a fairly longer duration—18 and 24 months, respectively. However, it is not clear whether the duration of the intervention had any impact on the efficacy of the intervention as other interventions in this review had a similar duration but yielded significant results [34]. The general consensus from the studies is that better ways of mobilizing students to take part in physical activities during recess should be employed to reduce physical inactivity. One way to increase physical activity could be transforming the school ovals and sports halls to put structures such as stationary bikes and other fitness equipment for students to use during recess. The prevalence of adolescent obesity in Australia warrants a review of the efficacy of the intervention programs to identify where the hindrances are.

There was weak evidence for the efficacy of reviewed intervention studies in reducing the prevalence of adolescent obesity based on BMI changes as only a third; 4 out of 12 studies resulted in significant reduction in BMI (references). These results are consistent with findings from other systematic reviews [46,47] where there were no statistically significant changes in BMI for both the intervention and control groups for most of the reviewed studies. Using BMI only as a measure of adiposity may not be accurate because BMI is not a reliable measure of excess body fat but a measure of excess body weight [48]. Other ways of measuring adiposity should be utilized in further studies for instance measuring fat mass index. A recent study [49] that used both BMI and fat mass index established that there was no relationship between leaner adolescents’ BMI with their fat mass index while a positive association between BMI and fat mass index was established for heavier participants. However, interventions focusing only on overweight or obese adolescents may appear discriminatory and obtain limited success, as Chen and Wilkosz [44] concluded that a non-discriminatory approach in interventions is more likely to yield success. Measuring adiposity using dual energy absorptiometry (DEXA) is costly, other measures such as using BMI and weight to height ratio have been found in one study [50] to be strongly correlated to body fat measured by DEXA. Future interventions should therefore use a combination of BMI and weight to height ratio since these measures are easy to use and affordable.

With regard to physical activity, one study [36] in this review provided strong evidence that incorporation of physical activity alone as an intervention has the potential to significantly reduce obesity [51]. The study also utilized three theoretical frameworks—the social cognitive theory, socio-ecological theory, and the health promoting schools framework—a possible reason why the intervention yielded positive results. The review findings confirm that the current status of physical activity among Australian adolescents is below the national standards [22,52]. The high prevalence of physical inactivity needs to be addressed in every state and territory through development and evaluation of physical activity policies, especially in Queensland and South Australia where a recent study [53] could not publicly identify these policies. It is generally agreed that physical activity plays a significant role in reducing obesity and maintenance of a healthy weight [46,54]. The findings of this review are crucial for policy makers, school leaders and the government to strive to reduce the prevalence of physical inactivity among Australian adolescents which has recently been escalated by the Covid-19 pandemic [18].

A quarter of the studies [31,40] that measured the level of consumption of sugar-sweetened beverages, fresh fruit and vegetables yielded significant results providing weak evidence for this type of intervention. These findings are partially consistent with a study [55] that systematically assessed the impact of multi-strategy nutrition education programs on the health and nutrition of adolescents. For instance, Meiklejohn, Ryan and Palermo [55] found significant dietary changes in the consumption of fresh fruit, vegetables and fat but not in sugar-sweetened beverage consumption. A possible explanation for the high consumption of unhealthy food by adolescents could be the choice of unhealthy fatty and sugary food available at the school tuck-shop and lack of role models. More intervention studies on nutrition behaviors are needed while schools and state governments need to promote healthy eating within the school environment.

Some of the reviewed studies employed teacher facilitation of nutrition programs, staff presence and changes in the food environment, but minimal changes were seen. This weak link between teacher involvement and modification of the food environment during the intervention is consistent with findings from past studies. For instance, a systematic review [56] also found that the seven programs reviewed demonstrated limited success in improving students eating behaviors. Another similarity between our findings and Godin, Leatherdale and Elton-Marshall [56] is that the knowledge about healthy eating significantly increased albeit with minimal impact on the actual eating behaviors of participants. The findings also suggest that there is a decline in healthy food choices by adolescents despite the knowledge gained on healthy eating. Possible reasons for minimal success of the intervention in yielding desirable results could be because of the disconnect between student dietary choices at school and dietary habits after school hours. This pattern warrants a more in-depth understanding of the dynamics of promoting healthy eating at this critical stage of development. A further exploration of teacher perspectives on the enablers and barriers to implementing intervention programs in school is recommended for future studies. More importantly, due to the many health implications associated with obesity, it is expedient to target preventive measures rather than a cure.

### Study Strengths and Limitations

To the best of our knowledge, this is the first review paper that focuses on the efficacy of Australian school-based interventions targeted at adolescent obesity. The findings of this study provide a roadmap to possible adjustments that may facilitate effective adolescent obesity interventions and directions for future research. Additionally, the reviewed studies were RCTs and quasi-experimental research designs and they were judged to be appropriate because they provide a high level of reliable evidence. Nonetheless, the findings of this study are limited by the inclusion and exclusion criteria. Some obesity intervention programs targeting adolescents were excluded because they were not implemented in a school setting. The study was also limited to an Australian context. However, the majority of studies were from NSW, and so it is conspicuous that there is a paucity of data from other states and territories—for example, only one study from QLD. It is uncertain whether our findings can be generalizable across Australia. In addition, the variability in the interventions implemented and outcomes measured, as stated earlier, prevented a meta-analysis of the findings. Finally, by limiting included articles to those published in peer-reviewed journals, we may have missed other high-quality studies that are published in non-peer-reviewed journals.

## 5. Conclusions

Interventions combining physical activity and dietary outcomes or physical activity with incorporated theoretical framework in the intervention design are much more promising. It is not clear if intervention duration has any impact on the efficacy of interventions. Additionally, research on intervention studies, perceptions of school stakeholders on adolescent obesity interventions and hindrances and enablers to the implementation of interventions across other states and territories other than NSW is needed to combat the adolescent obesity epidemic and achieve sustainable long-term impacts. A stronger voice from political leaders and the mass media is crucial in lowering the prevalence of adolescent obesity post Covid-19 restrictions.

## Figures and Tables

**Table 1:** Characteristics of included studies.

Authors and Year of Publication	Study Design and Duration	Region and Socio-Economic Status (SES)—Low, Medium, High, Not Indicated (NI)	Sample Size (*n*); Mean Age ± SD; Range of Participant Groups
1. Contardo Ayala et al., 2018 [35]	Quasi-experimental intervention trial 4–17 weeks (approximately 4 months)	VIC—Melbourne NI	(*n* = 88); 14.8 ± 1.7 years; boys and girls Intervention and control from 1 school
2. Dewar et al., 2013 [30]	Cluster Randomized Controlled trial 24 months	NSW Low SES	(*n*= 357); 13.2 years; adolescent girls from 12 secondary schools
3. Hollis et al., 2016 [36]	Cluster Randomized Controlled trial 12–24 months	NSW Low SES	(*n* = 1233); 12 years; Grade 7 boys and girls from 10 schools
4. Lubans et al., 2016 [37]	Cluster Randomized Controlled trial 18 months	NSW Low SES	(*n* = 361); 12.7 ± 0.5 years; boys from 14 schools
5. Lubans et al., 2011 [31]	Cluster Randomized Controlled trial 6 months	NSW NI	(*n* = 100); 14.3 ± 0.6 years; boys from 4 schools
6. Malakellis et al., 2017 [32]	Quasi-experimental using a longitudinal cohort follow-up after 3 Years	ACT High SES	(*n* = 656); 12–16 years; boys and girls, 6 schools: 3 controls, 3 interventions
7. Millar et al., 2011 [33]	Quasi-experimental using a longitudinal cohort follow-up 3 years	Barwon, South Western VICTORIA Low SES	(*n* = 2054); 14.6 years; boys and girls 5 intervention schools, 7 control schools
8. Morgan et al., 2012 [34]	Cluster Randomized Controlled trial 6 months	NSW Low SES	(*n* = 100, 14.3 years; boys intervention (*n* = 50), control group (*n* = 50)
9. Parrish et al., 2018 [38]	Two-arm parallel group randomized controlled trial 5 months	NSW NI	(*n* = 172); 14.7 ± 0.7 years; Grade 7 boys
10. Peralta et al., 2009 [39]	Randomized Controlled Trial 6 months	NSW—Sydney NI	(*n* = 33); 12.5 ± 0.4 years; boys Intervention and control
11. Smith et al., 2014 [40]	Cluster Randomized Controlled Trial 20 weeks-around 4.5 months	NSW Low SES	(*n* = 361); 12.7 ± 0.5 years; boys 14 high schools
12. Sutherland et al., 2016 [41]	Randomized Controlled Trial 24 months	NSW Low SES	(*n* = 1150); 12 yrs; Grade 7 boys and girls 10 secondary schools
13. Weeks and Beck., 2012 [42]	Cluster Randomized Controlled trial 8 months	QLD—Gold Coast SES not sighted	(*n* = 99); 13.8 ± 0.4 years, Grade 9 adolescents (*n* = 46) boys, (*n* = 53) girls One high school

ACT—Australian Capital Territory; NSW—New South Wales; QLD—Queensland; SES—Socio-economic status; Vic—Victoria.

**Table 2:** Intervention and outcome measures. Intervention characteristics included (e.g., location of study, age, sex, duration of study, type of study, intervention components, main objective, obesity-related outcomes (i.e., Body Mass Index (BMI), age and sex standardized Body Mass Index (BMIz), waist circumference, body weight, body fat, lean mass and energy expenditure), physical activity and nutrition outcomes and behavioral outcomes).

Authors and Study Design	Intervention Type and Main Objective/Aim	Intervention Details/Components	Main Findings (Intervention vs. Control)	Theory
Contardo Ayala et al., 2018 [35] Quasi-experimental intervention trial	Sedentary Behavior—use of height-adjustable desks to reduce classroom sitting time Aim: To investigate the impact of an intervention to reduce classroom sitting time on adolescents’ energy expenditure	A secondary school classroom was equipped with height-adjustable desks, posters promoting the health benefits of and strategies for breaking-up sitting time and desk stickers reminding students to periodically stand up. Classroom teachers participated in a professional development session. The intervention used the classroom equipped for the intervention 2–5 times per week while the comparison used a traditional classroom. A Sense Wear Armband was worn by the participants to measure EE.	BMI was similar in the two groups at baseline, at 4 weeks and at 17 weeks, (*p* > 0.05). Waist circumference was significantly lower (3.5 cm at 4 weeks and 2.6 cm at 17 weeks, respectively, *p* < 0.05), while energy expenditure was significantly higher in the intervention (*p* < 0.05) compared to the control group BF, BW, PA, SC, NT and LM were not measured.	Not sighted
Dewar et al., 2013 [30] RCT	Physical activity and sedentary behavior Aim: To evaluate the 24-month impact of a school-based obesity prevention program among adolescent girls living in low-income communities	NEAT Girls used: - lifestyle promoting strategies (e.g., walking to school) - lifetime physical activity (resistance training), - improvement of dietary intake - reduction in sedentary behaviors. Intervention components: enhanced school sport sessions, lunchtime physical activity sessions, nutrition workshops, interactive educational seminars, pedometers for self-monitoring, student handbooks, newsletters and text messages to reinforce and encourage targeted behaviors.	After 24 months, there were no significant effects on BMI (*p* = 0.353) and BMIz *p* = 0.178) while percentage body fat was significantly reduced (−1.96%, *p* = 0.006) in favor of the intervention group. There were no significant changes recorded for BMI and BMIz for the control group. No significant changes were observed for physical activity and screen time (*p* = 0.257 and *p* = 0.159), respectively. WC, BW, BF and PA not measured.	Social Cognitive Theory
Hollis et al., 2016 [36] RCT	Physical activity—using an accelerometer during waking hours AIM: To report whether the intervention impacted on adiposity outcomes (weight, BMI, BMIz) and whether any effect was moderated by sex, baseline BMI and baseline physical activity level, at 12 and 24 months	The “Physical Activity 4 Everyone” intervention operated under seven PA strategies categorized into Formal Curriculum (i.e., active lessons, Personal PA plans, Enriched sports), School Ethos and Environment (recess and/or lunchtime activities, supportive school PA policy). Implementation strategies included an in-school physical activity consultant 1 day per week, establishing leadership and support, teacher training, resources, teacher prompts and intervention implementation feedback to schools.	A significant impact of the intervention was recorded for BMI (*p* = 0.0116) and BMIz (*p* = 0.006). Moderate impact on weight (*p* = 0.0396) and BMI at 12 months, and weight, BMI and BMI z-score at 24 months in favor of the intervention group; *p*-value range (*p* < 0.01 to *p* < 0.02) whereas no changes were recorded for the control group. The trial had desirable long-term outcomes with 70% incorporated physical activity plans. WC, BF, LM, EE, PA, SC and NT not measured.	Social Cognitive Theory, Socio-ecological theory, Health Promoting schools Framework
Lubans et al., 2016 [37] RCT	Physical activity, nutrition and sedentary behavior—avoiding screen time Aim: To evaluate the sustained impact of the “Active Teen Leaders Avoiding Screen time” (ATLAS) obesity prevention program.	ATLAS intervention included teacher professional learning for readiness to deliver enhanced sport sessions (2 × 5 h workshops), provision of fitness equipment to schools (1 × pack/school valued at around USD 1500), researcher-led seminars for students (3 × 20 min), face-to-face physical activity sessions delivered by teachers during the school sport period (20 × approximately 90 min in addition to regular PE lessons), lunchtime physical activity leadership sessions run by students (6 × 20 min), pedometers for physical activity monitoring (17 weeks) strategies for reducing recreational screen time (4 × newsletters) and a purpose-built smartphone application (15 weeks).	No significant change for all measures except for screen time which was reduced (*p* = 0.003); not successful. NS for BMI (*p* = 0.656), BMIz (*p* = 0.485), Waist circumference (*p* = 0.549), PA (*p* < 0.05) and SSB (*p* = 0.561). BW, BF, LM and EE not measured.	Self-Determination Theory and Social Cognitive Theory
Lubans et al., 2011 [31] RCT	Physical activity, sedentary and nutritional behaviors. Aim: To prevent obesity among adolescents.	Components included school sports sessions, interactive seminars, lunchtime activities, physical activity and nutrition handbooks, leadership sessions and pedometer for self-monitoring. Focused on the promotion of lifestyle activities and lifetime activities and delivered over two school terms at no cost to the school or students. Participants completed self- and teacher-directed sessions. Teachers supervised student-led activities. Participants who completed the set activities were recognized and given certificates at school assembly of which about 50% of participants were awarded certificates.	There was a significant reduction in BMI for the intervention group (*p* < 0.001) and BMIz (*p* < 0.001); no changes for the control group. The intervention group also significantly reduced consumption of sugary beverages (*p* < 0.02), Significant reduction also observed in screen time (*p* < 0.05). NS changes for waist circumference and physical activity. Significant reduction in the number of participants classified as overweight or obese (*p* = 0.03). LM, EE and SC not measured	Social Cognitive Theory
Malakellis et al., 2017 [32] Quasi-experimental intervention trial	Nutritional and sedentary behaviors and physical activity—multiple initiatives OBJ: To reduce unhealthy weight gain by promoting healthy eating patterns, regular physical activity, healthy body weight and body size perception amongst youth; improve the capacity of families, schools, and community organizations to sustain the promotion of healthy eating and physical activity in the region.	The Fitness Improvement Lifestyle Awareness program (FILA) components included physical activity at school, active transport to and from school within 30 min walking distance. Key personnel—Principals, PE Teachers, students, representatives from ACT Health Directorate, ACT Education and Training Directorate and Nutrition Australia participated in a two-day workshop to develop the multi-component intervention. Participating intervention schools were given funds towards redeveloping the school environment to support nutrition and physical activity, fitness and sport equipment and for costs associated with presentations to intervention schools.	There was no statistically significant change in percentage body fat. Significant changes recorded for BMI (*p* < 0.05) and healthy eating awareness for the intervention group but no significant impact on the food environment. Significant increase in screen time (*p* < 0.02) for the intervention group and significant decrease for the comparison group. Significant fresh fruit and vegetables intake (*p* < 0.05) and physical activity (*p* < 0.05 during recess) Active transport—NS (*p* < 0.143). Two of the intervention schools showed a significant decrease in the prevalence of overweight and obesity (*p* < 0.05) and no changes for the control group. Body fat NS, WC, LM and EE were not measured.	Social Cognitive Theory
Millar et al., 2011 [33] Quasi-experimental intervention trial	Nutritional behavior, sedentary behavior and physical activity—multiple initiatives AIM: To report the outcome results of anthropometric indices and relevant obesity-related behaviors.	The intervention focused on 10 objectives, each comprising a variety of strategies delivered in schools through school project officers and student ambassadors. The aims were capacity building among officers and students, increase g awareness of project messages, evaluation, promoting water and reducing soft drinks, removal of soft drinks from vending machines, promoting healthy breakfast and consumption of fruit and vegetables, increasing healthiness of school food, promoting active transport and acceptance of body size and shape and finally increasing participation in organized sport. New equipment was installed, vegetable gardens constructed, vending machines removed, school food and water policies were introduced.	Statistically insignificant changes in BMI (*p* = 0.06), significant changes in BMIz (*p* < 0.03), body weight (*p* < 0.04), significantly less weight gained (740 g) as compared to the control group than the control and active transport significantly decreased (*p* < 0.01) in both the intervention and control group. Screen time increased for the intervention (*p* < 0.05) group as compared to the significant decrease in the control (*p* = 0.01.) NS changes for sugar-sweetened beverages, s snack food intake from take away shop and fruit drinks/cordial (*p* > 0.05), PA recorded NS (*p* < 0.05) Body fat recorded NS results for both groups (*p* = 0.58) as well as BMI (*p* < 0.06) WC, LM and EE not measured	Community-based capacity building approach
Morgan et al., 2012 [34] RCT	Physical activity Aim: To evaluate the effect of a school-based obesity prevention program and physical self-perception and key physical activity-related cognitions in adolescent boys from disadvantaged secondary schools	Components included enhanced school sport sessions with a focus on resistance training, physical activity and nutrition handbooks with home-based challenges, interactive seminars addressing key physical activity and nutrition behaviors, leadership principles and self-directed lunchtime exercise sessions. The boys participating were encouraged to become physical activity leaders in their school and accreditation was provided to students who complied with the program.	Two of the three intervention schools showed a significant decrease in the prevalence of overweight and obesity and increased physical self-worth, perceived physical condition, resistance training self-efficacy and physical activity behavioral strategies (*p* = 0.02), Body fat (*p* = 0.04). Significant results for all PE-related psychological outcomes (ranging from *p* < 0.01 to *p* = 0.04) and BMI (*p* < 0.001). WC, BW, LM, EE, SC and NT not measured.	Social Cognitive Theory
Parrish et al., 2018 [38] RCT	Sedentary Behavior—reduced sitting time Aim: To evaluate the feasibility, acceptability, and potential efficacy of a school-based intervention to reduce adolescent sitting time during the school day.	The intervention involved classroom and outdoor environment measures to break up and reduce the proportion of adolescent school time spent sitting measured by activPal monitors. The intervention was implemented by classroom teachers. Intervention schools were given stand-biased desks, free standing whiteboards and standing outdoor tables to be used for 30 min daily during the intervention.	No significant effect on body fat (*p* = 0.36) and BMI (*p* = 0.12). WC, BW, LM, EE, PA, SC and NT were not measured.	Not sighted
Peralta et al., 2009 [39] RCT	Physical activity promotion through Fitness, self-efficacy, self-esteem improvement, sedentary behavior through screen time reduction and nutrition behaviors Aim: To evaluate the feasibility, acceptability, and potential efficacy of a school-based obesity prevention program among adolescent boys with sub-optimal cardiorespiratory fitness.	The intervention focused on promoting physical activity through increasing physical self-esteem and self-efficacy, reducing time spent in small screen recreation on weekends, decreasing sweetened beverage consumption, and increasing fruit consumption and the acquisition and practice of self-regulatory behaviors such as goal setting, time management and identifying and overcoming barriers. Behavior modification techniques and use of incentives such as small footballs were used throughout the program.	No significant effects on all health outcomes measured for both the intervention and the control. Only 50% of intervention group attended lunchtime PA sessions. Weekday and weekend moderate and vigorous PA had insignificant results (*p* < 0.05). Fresh fruit (*p* = 0.18), BMI (*p* = 0.50), body fat (*p* = 0.30), sugar-sweetened beverage intake (*p* = 0.65) and waist circumference (*p* = 0.27). BW, LM, EE and SC not measured	Social cognitive theory
Smith et al., 2014 [40] RCT	Sedentary behavior through avoiding screen time, nutrition and physical activity Aim: To evaluate the impact of the Active Teen Leaders Avoiding Screen time (ATLAS) intervention for adolescent boys, an obesity prevention using smartphone technology.	The Active Teen Leaders Avoiding Screen time (ATLAS) intervention components involved teacher professional development, one fitness instructor, parent newsletter distribution, researcher-led seminars for students, enhanced 90 min school sports sessions, lunchtime physical activity mentoring sessions, smartphone app and website and also use of pedometers.	Significant effects recorded for screen time (*p* = 0.03) and intake of sugar-sweetened beverages (*p* = 0.01) but no significant effects on BMI (*p* = 0.84), waist circumference (*p* = 0.16) and body fat (*p* = 0.99). BW, PA, EE and LM not measured	Self-determination theory and social cognitive theory
Sutherland et al., 2016 [41] RCT	Physical activity Aim: To report the 12-month mid-point effect of a two-year multi-component physical activity intervention implemented in disadvantaged secondary schools.	The intervention operated under seven PA strategies categorized into the formal curriculum (i.e., active lessons, personal PA plans, Enriched sports), school ethos and environment (recess and/or lunchtime activities, supportive school PA policy) and community links (i.e., linking with parents, linking with the community). Implementation strategies included an in-school physical activity consultant 1 day per week. Trained research assistants were blinded to group allocation.	Overall, no significant effects on physical activity (*p* > 0.05) for both the intervention and control but there were significant differences noted within the intervention group; boys performed better (*p* = 0.02) in PA as compared to girls (*p* = 0.35). BMI, WC, BW, BF, LM, EE, SC and NT not measured.	Social cognitive and Social-ecological theory, Health Promoting Schools Framework
Weeks and Beck, 2012 [42] RCT	Physical activity-jumping regime Aim: To determine the effect of a twice-weekly, school-based, 10 min jumping regime on muscle and fat tissue in healthy adolescent boys and girls	An instructor coordinated and demonstrated all jumping activities such as jumps, hops, tuck-jumps, jump squats, stride jumps, star jumps, lunges and skipping. Participating students had to complete two sessions of 10 min jumps (approximately 300 jumps each session) each week. The control group undertook regular PE warm-ups and stretching directed by their usual PE teacher, and undertook brisk walking and light jogging for normal PE lessons.	No differences recorded for measurements at baseline and at 8 weeks between the intervention and control group. Significant fat loss (*p* = 0.10) and significant gain in lean tissue (*p* = 0.016) recorded for boys than girls in the intervention group. No significant effects were recorded for BMI (*p* = 0.810) and body weight (*p* = 0.398) for both of the intervention and control groups. Girls in the control group increased their external physical activity level (*p* = 0.003), while the intervention group and boys in both groups did not. WC, EE, SC and NT not measured	Not sighted

WC—Waist circumference; BF—Body fat; BW—Body weight; LM—Lean Mass; EE—Energy expenditure; PA—Physical activity; SC—screen time; NT—Nutritional behavior; NS—not significant; S—significant; M—moderate.

**Table 3:** Quality Assessment Tool (QATSDD) for included studies.

Author and Year	1	2	3	4	5	6	7	8	9	10	11	12	13	14	15	16	Total Score	% Total Score and Quality Rating
1. Contardo Ayala et al., 2018 [35]	0	3	3	2	2	3	3	2	3	n/a	1	1	n/a	3	3	3	32/42	76% M
2. Dewar et al., 2013 [30]	3	3	3	1	1	3	1	2	3	n/a	2	2	n/a	2	3	3	34/42	81% M
3. Hollis et al., 2016 [36]	3	2	2	2	3	3	1	3	3	n/a	1	2	n/a	3	3	3	35/42	83% H
4. Lubans et al., 2016 [37]	2	2	2	3	2	3	0	2	1	n/a	1	0	n/a	3	3	3	27/42	70% M
5. Lubans et al., 2011 [31]	3	3	3	2	2	2	1	2	2	n/a	2	2	n/a	2	3	3	32/42	76% M
6. Malakellis et al., 2017 [32]	2	3	2	2	2	2	0	1	2	n/a	1	2	n/a	3	2	2	24/42	61% M
7. Millar et al., 2011 [33]	3	2	3	1	1	3	0	2	2	n/a	1	1	n/a	2	3	3	27/42	64% M
8. Morgan et al., 2012 [34]	3	3	2	2	1	2	0	2	2	n/a	2	2	n/a	1	3	3	26/42	62% M
9. Parrish et al., 2018 [38]	0	3	2	3	2	2	0	2	1	n/a	1	1	n/a	3	3	3	23/42	55% M
10. Peralta et al., 2009 [39]	3	3	2	2	2	2	3	2	1	n/a	1	2	n/a	3	3	3	32/42	76% M
11. Smith et al., 2014 [40]	3	2	3	2	2	2	2	3	2	n/a	1	2	n/a	3	3	3	33/42	79% M
12. Sutherland et al., 2016 [41]	3	2	3	3	3	0	2	2	0	n/a	1	2	n/a	3	3	3	30/42	71% M
13. Weeks and Beck, 2012 [42]	0	3	3	3	3	2	0	2	2	n/a	1	1	n/a	1	3	3	27/42	70% M

QATSDD—Quality Assessment Tool for Studies with Diverse Designs (KEY: 0 = not at all, 1 = very slightly, 2 = moderately, 3 = complete, n/a = not applicable, L = Low quality; below 50%, M = Medium quality; 50%–80%, H = High quality; over 80%). The details of column title 1–16 can be found in the Appendix A.

## Appendix A

**Item Number**	**QATSDD Criteria**
1	Explicit theoretical framework
2	Statement of aims/objectives in main body of report
3	Clear description of research setting
4	Evidence of sample size considered in terms of analysis
5	Representative sample of target group of a reasonable size
6	Description of procedure for data collection
7	Rationale for choice of data collection tool(s)
8	Detailed recruitment data
9	Fit between stated research question and method of data collection (Quantitative only)
10	Fit between research question and format and content of data collection method (Qualitative only) EXCLUDED
11	Fit between research question and method of analysis (Quantitative only)
12	Good justification for analytical method selected
13	Assessment of reliability of analytical process (Qualitative only); EXCLUDED
14	Strengths and limitations critically discussed
15	Evidence of use involvement in design
16	Statistical assessment of reliability and validity of measurement tools
